# An assessment of a health education program addressing overweight, obesity and lifestyle modification in Ghana

**DOI:** 10.1371/journal.pone.0333625

**Published:** 2025-11-13

**Authors:** Jane Barnes, Sandra Boatemaa Kushitor, Millicent Ofori Boateng, Edward Kofi Sutherland, James Avoka, Stephen Manortey

**Affiliations:** 1 Department of Community Health, Ensign Global University, Kpong, Ghana; 2 Department of Food Science and Centre for Sustainability Transitions, Stellenbosch University, Stellenbosch, South Africa; 3 Lower Manya Municipal Health Directorate, Ghana Health Service, Odumase-Krobo, Ghana; University of Health and Allied Sciences, GHANA

## Abstract

**Background:**

Obesity is a significant risk factor for several comorbidities, including type II diabetes, cancer, and cardiovascular diseases. Recent studies suggest an increase in the prevalence of overweight and obesity among rural residents. Yet interventions to create awareness and promote lifestyle modifications are limited in rural areas.

**Aim:**

The aim of this paper is to assess the effect of a nutrition education intervention in enhancing overweight and obesity knowledge and lifestyle modification in a rural area in the Eastern Region of Ghana.

**Methods:**

The study employed an exploratory sequential mixed-methods design and implemented a community-based intervention that involved nutrition education sessions, including songs, food demonstrations, and community engagement called the Oklebenor Awareness Program. Baseline and end-line surveys and in-depth interviews were conducted on the socio-demographic characteristics, participants’ knowledge, attitudes, and behaviours regarding overweight and obesity. The quantitative data were analysed using means, frequencies and T-test. The in-depth interviews were analysed using thematic analysis (n = 22).

**Results:**

The average attendance during the lessons was 45 participants. About 50% of the respondents attended 4 lessons. The participants reported the Oklebenor Awareness Program as their main source of knowledge on overweight and obesity (n = 21). The mean score on the Obesity Risk Knowledge Score-10 increased from 5.3 at baseline to 6.6 at endline (mean change = 1.39, p = 0.001). Risk factors of overweight and obesity reported by the participants included unhealthy eating, physical inactivity, alcohol consumption and intentional weight gain. Lifestyle changes reported by the participants due to the intervention included increased dietary diversity by including legumes, fruits and vegetables in their meals and physical activity.

**Conclusion:**

The nutrition education intervention enhanced participants’ knowledge of overweight and obesity and promoted dietary diversity and physical activity. Incorporating culturally sensitive approaches and involving families and community resources contributed to the success of the intervention. The findings highlight the need for health education programs to address the rising prevalence of overweight and obesity in rural areas.

## 1. Introduction

The prevalence of overweight and obesity has increased in most countries [1]. According to the NCD Risk Collaboration, the prevalence of overweight and obesity between 1990 and 2022 increased 89% for women and 73% for men globally [1]. Obesity increases the risk of cardiovascular diseases, type 2 diabetes, hypertension and other chronic non-communicable diseases [2], thus increasing household healthcare expenditure. In Ghana, obese individuals spend three times more on healthcare ($132) than those with a normal weight ($35) [3]. In South Africa, about 5.38% of government health spending, or 0.67% of GDP, was on obesity related healthcare [4].

Overweight and obesity have long been linked to urbanisation [5–7]. However, recent studies suggest that obesity is increasing in rural areas [8]. For example, the prevalence of overweight and obesity among rural resident women increased from 4% in 2014 to 9% in 2022 [9,10]. In rural areas, the increased penetration of sugar-sweetened beverages, fewer opportunities for leisure-time physical activity, and scarce healthcare services have been associated with overweight and obesity [11]. Furthermore, low-income individuals in rural areas may lack access to resources that can enable weight reduction strategies. As a result, government policies have stressed the importance of tackling lifestyle risk factors such as insufficient physical activity, poor dietary habits and quality [8].

Health education has been identified as a key intervention for addressing overweight and obesity by improving knowledge about healthy eating habits and physical activity [12,13]. It is one of the recommendations of the International Obesity Taskforce (IOTF) Framework for preventing obesity [14]. In Africa, most obesity interventions with behavioural (such as healthy eating, physical activity) and anthropometric outcomes are among children [15]. The interventions are also limited geographically since they were concentrated in Tunisia, South Africa and Uganda [16]. Most of the interventions are community and school-based and incorporate mobile technology. However, a systematic review indicated that these interventions had no significant effect on dietary behaviour due to limited resources [16]. Although ownership of mobile phones has increased, mobile network connectivity has consistently been an obstacle to mhealth interventions, especially in rural areas [17,18].

Nearly half of Ghanaian women are overweight or obese, according to the 2022 Ghana Demographic and Health Survey. Although the prevalence of overweight and obesity is high in Ghana’s urban regions, it is progressively rising in rural areas. Between 2014 and 2022, the prevalence of overweight and obesity in rural areas increased from 8% to 12% among men and 29% to 37% among women, respectively [9,10]. However, there is limited evidence about the effects of community-based interventions for increasing knowledge and lifestyle modification in Ghana for the prevention and management of overweight and obesity in rural areas.

In 2019, the IDRC funded a multisectoral nutrition program to support women agricultural entrepreneurs in the Eastern Region of Ghana. The project was the Scaling Up Women’s Agripreneurship Through Public-Private Linkages to Improve Rural Women’s Income, Nutrition, and the Effectiveness of Institutions in Rural Ghana (*LinkINg Up*) [19]. The prevalence of overweight and obesity from the *LinkINg Up* baseline data was 10% higher than the national average in 2014 [20]. Therefore, the stakeholders requested for an intervention to increase overweight and obesity awareness. In 2022, a facilitator guide on overweight and obesity was designed collaboratively with the Women in Agricultural Development (WIAD) of the Department of Agriculture and Nutrition Officers from the District Health Directorates [20]. The implementation of the facilitator guide was called the Oklebenor Awareness Program.

The Oklebenor Awareness Study implemented and evaluated a community-based intervention to increase awareness, prevention and management of overweight and obesity in the Lower Manya Krobo Municipality of the Eastern Region of Ghana. The intervention promoted healthy eating and physical activity through integrated community engagement. This paper aims to describe the effect of the intervention on respondents’ knowledge and awareness of overweight and obesity, and lifestyle modifications. Specifically, we assessed: 1) participants’ experiences of the intervention, 2) their understanding of risk factors associated with overweight and obesity, and 3) changes in their intake of fruits and vegetables as well as their levels of physical activity.

## 2. Materials and methods

### 2.1. Study design and setting

This study used an exploratory sequential mixed-method design [21]. The quantitative data were collected first, followed by qualitative data for deeper interpretation. The quantitative data was a quasi-experiment with pre- and post-intervention data collection in December 2022 and August 2023. It was a follow-up of a broader initiative called the *LinkINg Up* Initiative, which involved six Farmer-Based Organisations (FBOs) within three districts in the Eastern Region of Ghana [19]. The baseline data of *LinkINg Up* reported a high prevalence of obesity similar to that of urban areas. Therefore, at the request of the local government officers, a facilitator guide was developed through a multisectoral process on obesity awareness [20].

The FBO in Ayermasu in the Lower Manya Municipality was the study unit. Ayermasu is a rural community characterised by lush vegetation and small-scale agriculture. The primary livelihood for most of the community members is subsistence farming, focusing on crops such as maize, cassava, and plantains. Community health workers in Ayermasu run a child welfare clinic focused on the health of children under five and their mothers. This means that most women who do not have children in this age group do not have access to these community health services.

The methodology and results of this study are reported using the checklist for reporting group-based behavioural change interventions [22]. Ethical approval for the study was granted by the Ethics Review Committee of Ensign Global University (EKSUTH/A67/2020/06/007). Written informed consent was obtained from all facilitators and participants. Data were collected anonymously. The survey and in-depth interviews were conducted privately, and only the research team had access to the socio-demographic details, primarily for recording meeting attendance during the intervention days.

### 2.2. Oklebenor awareness program

The ‘Facilitator Guide for Nutrition Education on Overweight and Obesity’ developed by Kushitor and Colecraft [20] was used for the community nutrition education intervention. The guide consists of five lessons aimed at raising awareness about obesity and developed based on the stages of change theory. Four documents informed the content of the guide: 1)Nutrition Facts for Ghanaian Families, 2) the Dietary and Physical Activity Guidelines for Ghanaians, 3) the information sheet of the obesity risk knowledge scale (ORK-10), and 4) the WHO materials on overweight and obesity and physical activity. Two consultants also contributed to the guide’s content. Consultant 1 is a Human Nutrition and Nutrition Education Lecturer at the University of Health and Allied Sciences and a trained FAO nutrition educator with expertise in developing nutrition education materials. Consultant 2 was an MSc student in Human Nutrition at McGill University with research expertise in overweight and obesity among rural farmers in Ghana. The guide was validated by nutrition and Women in Agriculture Development officers from the Upper Manya Krobo District Assembly, Yilo Manya Kro Municipal Assembly and the Lower Manya Krobo Municipal Assembly. Details on developing the guide can be found in Kushitor and Colecraft [20].

**Fig 1:**
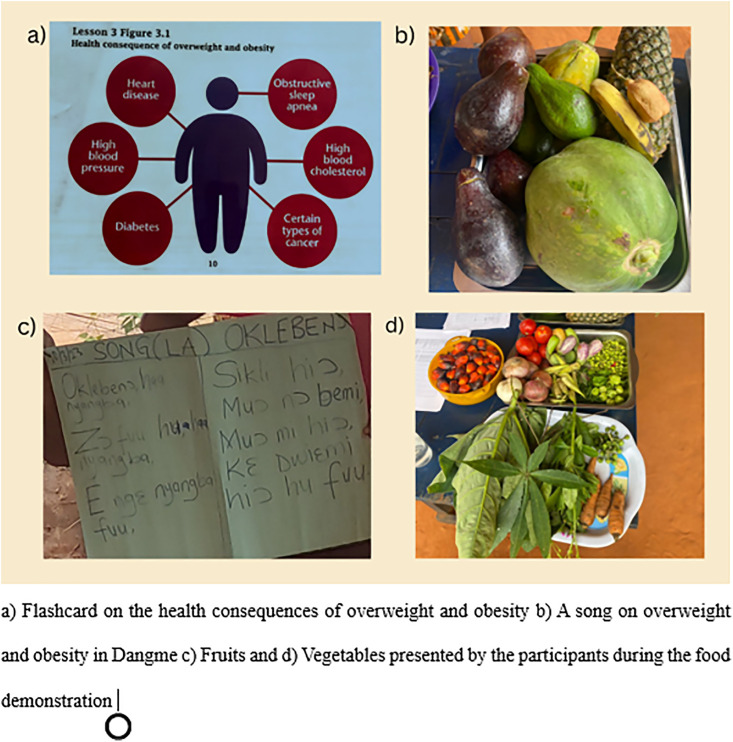
Pictures depicting intervention activities (about here).

The Municipal Director of Health Services recommended two nutrition officers, two health promotion officers, a midwife and a mental health officer as facilitators for the intervention. The officers could speak the local Dangme language and had previously worked in the community. These facilitators were trained before the intervention. Some agreed-upon keywords in the local dialect were identified to create a linguistic diary; some were ‘mi joor mi’, meaning bodyweight, and ‘oklebenor’, meaning obesity. The linguistic diary was used to consistently deliver key messages throughout the health education. For example, the facilitators would use ‘oklebenor’ anytime they were to say obesity. Also, the songs were translated into Dangme. The trained local officers were the main facilitators for the intervention. The intervention was introduced to the participants after the facilitators did a self-introduction. Other members, including the researchers, were also introduced to establish rapport between researchers, facilitators, and participants. The intervention participants were taken through the five lessons in a fortnightly education session from 2nd March 2023–14th April 2023 (Fig 1). The sessions were interactive. We had discussions with flashcards, role-plays and group activities. The participants were also allowed to ask questions during and after the lessons. The secretary of the FBO, the community leader, and the queen mother oversaw the coordination of the group before every meeting.

Lesson 1 defined overweight and obesity using the Obesity Risk Knowledge Scale (ORK-10) information sheet and Stunkard Figure Rating Scale, highlighting the prevalence and causes of obesity [23,24]. Lesson 2 explored body image preferences, cultural norms, and their connection to unhealthy behaviours. Lesson 3 covered the consequences of overweight and obesity, including health, economic, and social impacts. Lesson 4 focused on achieving food variety through the four-star diet. The lesson is expected to be followed up with a food demonstration. The food demonstration session took participants through food preparation with locally available ingredients that meet the four-star diet principle. The participants provided local ingredients from their farms, backyards and kitchens. Lesson 5 is a session on physical activity. This lesson was presented on the same day as Lesson 4, due to the unavailability of the farmers for the subsequent meeting schedule. The intervention was administered to all members who attended the FBO meeting during each lesson. The research team also recorded attendance for participants recruited at baseline and other FBO members present at each lesson.

Songs were taught during Lessons 3, 4, and 5 in the local language. A song on overweight and obesity, its causes, and consequences was taught during lesson 3 in Dangme. Lesson 4 had a song about the ‘Four-star diet’ while Lesson 5’s song was on physical activity (also taught in Dangme). During the education sessions, various local fruits (e.g., bananas, watermelon, and oranges) were served as snacks and healthy local meals for lunch. This was part of efforts to inculcate the habit of fruit consumption among the study population. The participants’ anthropometric measures were also collected during the session. Body weight and height were collected once, but blood pressure was measured at each session for all participants. Some participants were not part of the baseline recruitment, but because the sessions were conducted during the weekly FBO meeting, the lessons were provided for all present members. The weekly attendance was between 58 and 50, averaging 45.

### 2.3. Data collection

#### 2.3.1. Baseline and endline quantitative data.

**Fig 2:**
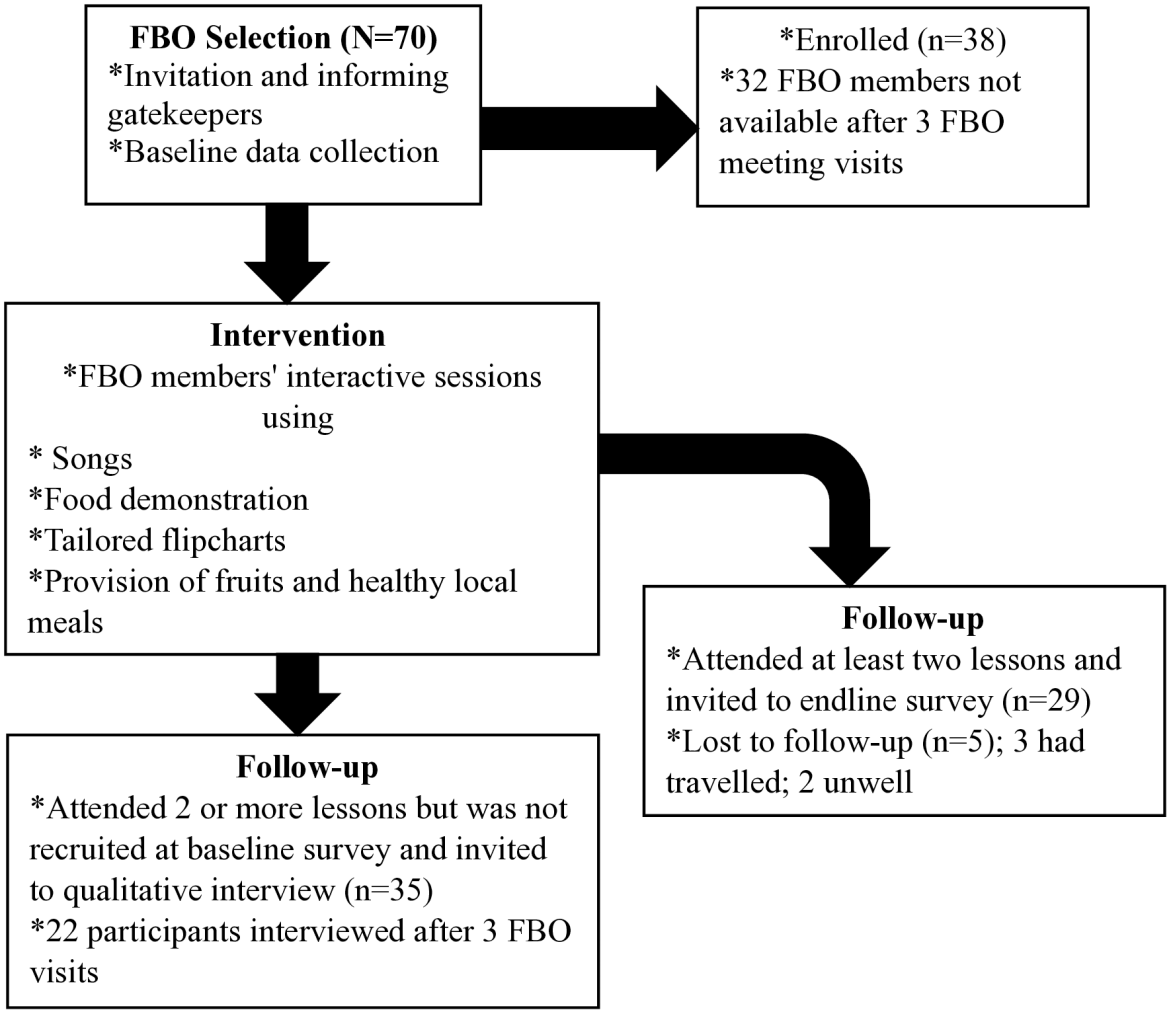
Flow chart of Oklebenor awareness study (about here).

According to the FBO secretary, there were 70 registered members as of August 2023. The study team aimed to recruit all 70 members (Fig 2). After attending three bi-weekly visits during FBO meetings, the team recruited 38 members at baseline. The baseline data collection included socio-demographic information such as age, sex, educational level, occupation, and marital status. Their knowledge of overweight and obesity, including their understanding of the risk factors, consequences, and preventive strategies, was assessed using the Obesity Risk Knowledge-10 scale (ORK-10) before and after the intervention. The questionnaire was administered using the KoboToolbox, and it was interviewer-led. Additionally, data on participants’ weight and height for BMI, and blood pressure were collected by trained research assistants from the Public Health Unit of the Health Directorate using the World Health Organisation (WHO) STEPwise approach to NCD risk factor surveillance (STEPS) guidelines [25]. Respondents who participated in at least two lessons met the inclusion criteria for the endline survey (n = 29); 5 out of the 29 were lost to follow-up.

#### 2.3.2. Endline qualitative data.

Semi-structured interviews were conducted with 22 FBO members who were not part of the baseline quantitative data but participated in 2 or more lessons. These participants were purposely selected based on their attendance. The data from the attendance sheet we took each week indicated that 35 participants were not recruited at baseline but had attended two or more lessons. We invited all 35 participants to the qualitative interview via telephone calls and community visits. After three visits spanning a period of six weeks, we were able to interview 22 of the participants.

The facilitators and trained research assistants from the Health Directorate conducted the interview using an interview guide. The interview guide was structured into five sections: awareness and content of the intervention, causes and consequences of overweight and obesity, prevention and management, sources of knowledge, and willingness to share their knowledge. The face-to-face interviews lasted between 12 and 20 minutes and were audio recorded and transcribed verbatim for analysis.

### 2.4. Data analysis

#### 2.4.1. Quantitative data.

The primary outcome of this study is the increase in obesity knowledge measured using the ORK-10. Within respondents, changes in ORK-10 were computed for each respondent, as the difference in their scores between baseline and endline.


Change in ORK−10 score=ORK−10 score endline – ORK−10 score baseline

We used frequency distribution and percentages to describe the socio-demographic variables and ORK-10 score. The socio-demographic variables included in the study were age, sex, marital status, educational level, and occupation. Sex was categorised as male or female. Marital status was categorised into two categories: currently married and currently not married (which included never married, divorced, and widowed). The participant’s level of education was none, primary and Junior High School (JHS). We excluded ethnicity and religion from our analysis because all the respondents were Krobo and Christians, respectively. The effect of the intervention on obesity knowledge was measured using a paired T-test among the 24 respondents with endline and baseline data.

#### 2.4.2. Qualitative analysis.

The analysis took place once all the interviews had been conducted, transcribed, and subjected to quality checks. Three transcribers with Krobo and English competence transcribed the interviews verbatim into English. The intervention facilitators were asked to listen to the audio files and read the transcripts concurrently. JB, SBK, and MOB cross-checked the transcripts with the linguistic diary developed during the facilitator training and reconciled conflicting narratives.

The data were analysed by JB, SBK, and MOB, as well as three research assistants with expertise in qualitative research methodology using thematic analysis in Dedoose [21]. The analysis covered the personal history of the participants and their awareness of the intervention. The analysis was started by reading transcripts to understand the data and identify emerging codes and themes. Inductive codes developed from obesity literature guided the analytical framework. The second stage of the analysis involved identifying the linkages between codes, themes, and appropriate respondent quotes.

Saturation was measured using code frequency count [22]. Thirty-four codes (7.6%) were identified from the first transcript. The six coders applied these initial codes to the next five transcripts. A total of 103 (83.7%) new codes were identified within the first six transcripts—the next set of five transcripts generated only 20 new codes. The remaining seven transcripts added four codes, making a cumulative total of 127. A total of 123 codes were realised after merging some of the codes.

#### 2.4.3 Integration of analysis and results.

We used an embedding approach to integrate the quantitative and qualitative results [26]. After analysing the quantitative data, we observed that the participants’ knowledge of obesity had increased. Thus, the qualitative data explored the participants’ experiences during the intervention and the mechanisms that facilitated this increase in knowledge. The qualitative results are presented to validate and substantiate the quantitative findings and highlight the process that resulted in the change in obesity awareness.

## 3. Results

### 3.1. Socio-demographic characteristics of respondents

A total of 38 members of the FBO participated in the study during the baseline, and 24 in the endline survey (Table 1). At the baseline, the largest proportion of participants (55%) belonged to the 25–49 age group. However, this dropped to 48% at the endline. The sex distribution indicated that females were the majority among participants (62% at baseline and 71% at endline). In terms of level of education, the JHS category held the largest share at baseline (47%) and endline (46%). Farmers comprised the largest portion at baseline (47%) and endline (67%). Half of the participants attended the lessons (50%). The qualitative sample had more men (59%) than women (41%), and about a third were within the 50–75 age group (59%). Most of them attended all the lessons (68%). About a third of the sample were overweight (53%) or obese (5%). The participants who dropped out of the study (36%) differed from those who did not. They were mainly young women traders of normal weight. They may not have had the time to attend the meeting due ot their trading activities. Most traders sell fresh produce at a nearby market on Saturdays, typically using Fridays to harvest and gather their goods. The intervention meeting occurred on a Friday. Among the participants, aged 50–75, who dropped out of the study, three had travelled, one was unwell during the follow-up survey, and one did not attend the lessons.

**Table 1:** Characteristics of study participants, endline and baseline.

Variables	Quantitative Data			Qualitative Data
	Baseline	Endline	Dropout	Endline
	n(%)	n(%)	n(%)	n(%)
**Age**				
25-49	21(55.3)	11(47.8)	9(64.3)*	9(40.9)
50-75	17(44.7)	12(52.2)	5(35.7)	13(59.1)
**Sex**				
Female	24(61.5)	17(70.8)	10(71.4)*	8(36.4)
Male	15(38.5)	7(29.2)	4(28.6)	14(63.6)
**Level of Education**				
None	5(13.2)	4(16.7)	6(42.9)	6(27.3)
Primary	11(28.9)	7(29.2)	1(7.1)	6(27.3)
JHS	18(47.4)	11(45.8)	5(35.7)	8(36.4)
SHS and above	4(10.5)	2(8.3)	2(14.3)	2(9.0)
**Occupation**				
Farmer	17(44.7)	16(66.7)	4(28.6)*	21(95.5)
Trader	16(42.1)	6(25.0)	9(64.3)	1(4.5)
Unemployed	5(13.2)	2(8.3)	1(7.1)	0(0.0)
**BMI**				
Normal	16(42.1)	9(37.5)	12(85.7)*	
Overweight	20(52.6)	7(29.2)	2(14.3)	
Obese	2(5.3)	8(33.3)		
**Attendance at the lessons**				
0			2(14.2)	
1			7(50.3)	
2		7(29.2)	2(14.2)	3(13.6)
3		5(20.8)	3(21.3)	4(18.2)
4		12(50)		15(68.2)
**Total**	**38(100)**	**24(100)**	**14(100)**	**22(100)**

*p-value between endline and dropout participants is < 0.005

### 3.2. Changes in ORK-10 scores between endline and baseline: Comparing Differences in Obesity Knowledge

Tables 2 and 3 present the scores on each item and the mean score of the ORK-10 scale. There was a significant increase in knowledge between baseline and endline. The proportion of participants who knew that a person with a ‘beer-belly’ shaped stomach has an increased risk of diabetes increased from 70% to 96%. The participants who knew that obesity increases the risk of getting bowel cancer increased from 48% to 87%, with a difference of 39% (p = 0.001). However, two questions saw a decrease in correct answers, though insignificant. The proportion of those who knew that obesity is a risk for black people reduced from 52% to 43% (p = 0.575). On average, the ORK 10 score was high (p = 0.001) with a mean difference of −1.39 at endline.

**Table 2:** ORK-10 correct scores of respondents at baseline and endline.

Obesity knowledge	Baseline (%)	Endline (%)	P-value
A person with a ‘beer-belly’ shaped stomach has an increased risk of getting diabetes	70.0	96.0	0.011
Obesity increases the risk of getting bowel cancer.	48.0	87.0	0.001
An obese person who gets diabetes needs to lose at least 40% of their body weight for clear health benefits.	39.0	26.0	0.492
Obese people can expect to live as long as non-obese people.	57.0	70.0	0.377
Obesity increases the risk of getting breast cancer after the menopause.	35.0	74.0	0.009
Obesity is more of a risk to health for white people than it is for black people like Ghanaians.	52.0	43.0	0.575
There is no major health benefit if an obese person who gets diabetes loses weight.	57.0	100.0	0.001
Obesity does not increase the risk of developing high blood pressure.	52.0	70.0	0.000
It is better for a person’s health to have fat around the hips and thighs than around the stomach and waist.	48.0	87.0	0.001
Obesity increases the risk of getting a food allergy.	43.0	52.0	0.539

**Table 3:** Difference in mean score on ORK-10 between baseline and endline.

Variable	Range	Mean	Std. err.	Std. dev.	95% CI		P-value
ORK-10 baseline	2-9	5.35	0.386	1.725	4.543	6.157	0.004
ORK-10 endline	4-8	6.6	0.255	1.142	6.065	7.135	
diff		−1.25	0.416	1.860	2.121	−0.379	

### 3.3. Participants’ experiences of the Oklebenor awareness program

The findings were grouped into three global themes: knowledge of overweight and obesity, the effects of the intervention on knowledge of overweight and obesity, and lifestyle modification.

#### 3.3.1. Knowledge of overweight and obesity.

This section reports on the causes, consequences and prevention of overweight and obesity. According to the participants, obesity is primarily caused by unhealthy eating and physical inactivity, alcohol consumption, intentional weight gain, drug use, and genetics in that order. They also reported that addressing these lifestyle behaviours is the primary approach to preventing obesity.

***Unhealthy eating and physical inactivity.*** Unhealthy eating was defined as the type of food and the quantity consumed (Table 4). While fatty and oily foods increased the risk of obesity, the consumption of green leafy vegetables like kontomire (taro leaves) and turkey berries and staples like plantain, was regarded as obesity prevention food items. These items were to be consumed in moderation. The quotes below represent these themes:

**Table 4:** Typology of the causes and prevention of overweight and obesity.

Components of eating habits	Food items and habits that cause obesity	Food items and habits that prevent obesity	Consequences of overweight and obesity
Type of food	Sugary food	Green leafy vegetables	Poor mental health
	Meat	Vegetables	
	Oily foods	Tubers	
		Fruits (e.g., banana, avocado pear, oranges)	Inability to work
		Legumes (beans)	Diabetes
Time of meals	Eating late in the evening		Hypertension
Portion sizes	Too much food at a time	Mindful eating	
Physical activity	Sitting in one place for long time	Exercise daily	
Alcohol intake	Too much alcohol		
Weight gain medication	B-complex vitamins and other medications

“*When you eat and refuse to exercise, you can have obesity. We need to be mindful of what we eat so that we don’t become obese.”* [R13]

*“Eating too much oily or fatty foods can make you obese. And also, they said what we eat sometimes can make us obese, such as sugary foods.” [*R7]

***Other causes of overweight and obesity.*** Participants mentioned that alcohol consumption contributes to overweight and obesity as suggested by a participant: *“Alcohol, too much drinking of alcoholic drinks can cause obesity”* [R11]. Other statements suggested there is a genetic predisposition to overweight and obesity in that people are likely to become overweight or obese if there is a family history of such.

***Consequences of obesity.*** The consequences of overweight and obesity reported by the participants included health challenges, psychological stress and inability to work:

*“We were taught that, when you’re obese, it brings health problems.”* [R7]

*“It brings about quick-temperedness in a person because we won’t be able to do the things we want to do.”* [R3]

#### 3.3.2. Effect of the intervention on their knowledge of overweight and obesity.

To answer this question, we asked the participants to indicate the source of their knowledge on obesity and to remember the lyrics of the three songs taught during the lessons. The dominant source of knowledge on overweight and obesity was the Oklebenor Awareness Project (n = 21), followed by mass media (n = 3), and community health nurses (n = 2). Other sources of information were the church (n = 2), health facility (n = 1), and school (n = 1). Some respondents had this to say:

*“I learnt about all these from the Oklebenor lessons held here.”* [R9]

*“Oh, it’s only your team, madam, I haven’t heard this from anywhere else.”* [R5]

Three songs on physical activity, the cost of being overweight or obese, and the four-star diet were taught during the intervention, respectively. Nineteen respondents mentioned they could remember the songs. Seven could sing one song or two songs. None of the respondents could sing all three songs. The song sung the most was the one about the consequences of being overweight or obese.

*“The obesity song that talks about the consequences of obesity.”* [R21]

*“I remember the song [respondent sang the oklebenor song well to the end].”* [R5]

Respondents related the effects of the intervention on their knowledge. According to them, the community is now aware of overweight or obesity (n = 15). They also reported changes in their diets and those of their families (n = 16), and physical activity levels (n = 11). The participants reported that the intervention has significantly impacted the community. The songs are sung when they meet at social gatherings.

*“There is a change, usually when we meet, we sing the song to remind ourselves of the problems associated with obesity.”* [R20]

#### 3.3.3. Effects of the Oklebenor Awareness Program on lifestyle changes.

***Dietary changes.*** The practice of dietary diversity dominated the dietary changes (n = 15). Some respondents reported adding beans to their stews and soups, eating more fruits and vegetables, and adding more stews or soups to their staple foods. This reflected the topic of the four-star diet, which was taught through songs and food demonstrations. Some responses indicated that households are now discussing healthy eating more frequently. The discussions highlighted mindful eating, increased fruit consumption, and reduced late meals and overeating.

*“The program has changed me, I do exercise after eating now. Initially, we only eat one-way [referring to limited dietary diversity] kind of food but now we try to get all the stars in our meals.”* [R3]

*“Oh, for example, now when I am about to eat yam, I add avocado pear to it as compared to previous times.”* [R3]

*“One thing I have learned is that I no longer eat late at night and, after eating I try to exercise. I also eat fruits.”* [R2]

***Physical activity.*** This theme provided insights into how participants responded to incorporating physical activity into their daily activities after participating in the intervention program. The responses could further be categorised under: increased post-meal exercise (n = 8) and family engagement in physical activity (n = 3). Several participants noted a change in their behaviour toward physical activity. One participant reported, “*I no longer sit at one place after my meals; I try to engage in exercise, and I see my family do the same nowadays.*” This was echoed in several other ways by other participants:

*‘It has changed me and now I do exercise after eating and also at first, we only eat one-way kind of food, but now we try to get all the stars [referring to the four-star diet] in our meals.”* [R3]

## Discussion

This study examined the effect of a community educational program on overweight and obesity knowledge among residents in a rural area in Ghana. Using a mixed-methodology study design comprised of a quasi-experimental methodology and in-depth interviews, the intervention was administered to members of the Ayermasu FBO. Participants in the FBO received context-specific lessons on the causes and consequences of obesity, a four-star diet food demonstration and physical activity. Eight months after the baseline survey, participants in the intervention had improved knowledge of overweight and obesity, and some had reported lifestyle changes.

The study also observed that the participants in the survey had improved knowledge of overweight and obesity after the intervention. These improvements could be because the participants knew they were under observation and might have responded favourably, or because of social desirability. However, the findings suggest otherwise, that the change observed in the mean score of the ORK-10 can be attributed to the intervention. The significant difference between the mean ORK-10 at baseline and endline indicates that without the intervention, the improvement in obesity knowledge might not have been recorded. Also, nearly all the qualitative participants reported the intervention as their source of knowledge on overweight and obesity. Following the intervention, participants reported positive changes in their behaviour. The participants reported engaging in post-meal exercises and incorporating diverse foods into their diets. This suggests that the intervention increased knowledge, translating into practical actions.

The participants in this study displayed varying levels of awareness and knowledge about overweight and obesity. The recognition of fatty foods as contributors to obesity highlights the importance of nutritional knowledge in influencing dietary choices. The mention of specific local foods like “kontomire” and “plantain” to prevent obesity underlines the cultural relevance of dietary recommendations. Furthermore, the incorporation of songs and food demonstrations during the intervention contributed to disseminating knowledge about obesity prevention and healthy eating habits. Using songs translated into the Dangme language and local foods in the intervention aligns with the assertion that culturally sensitive approaches are more likely to resonate with the target audience and elicit positive responses [27]. It has been confirmed in social and behavioural change education that music and demonstration reinforce and sustain learning [28].

Those who dropped out of the study were predominantly young women traders with a normal weight (refer to Table 1). In farming communities, young women usually take the produce to market centres, which sometimes can last days. This implies that they will not be available for the FBO meeting days. Also, most of them were of normal weight and may not have perceived the lessons as applicable to them.

There are several strengths to this study. Firstly, the study was grounded in principles of community engagement. The ability to engage and involve the local health department, chiefs, and leaders of the FBO helped foster co-production, trust and local contextualisation of the research. During the study period, the average attendance was 45 out of an FBO of 70 members (64%). To the best of our knowledge, the Oklebenor Awareness Program is the first of its kind to engage community members on overweight and obesity consistently for such a long time in Ghana. The high attendance rate observed in this research indicates that the participants were pleased with the program and that the material presented benefited them. Also, a key strategy behind the intervention design was to help the health department implement the program to ensure its sustainability. Thirdly, the study combined quantitative and qualitative research methods to explore the increase in knowledge and the experiences related to the intervention. The use of both methods made up for the limitations of each and also the small sample size.

However, this research has some limitations. The relatively small sample size of the FBO (n = 70) affected the sample for the quantitative component. Recruiting respondents for the study was challenging because they were mostly on the farm, except during FBO meeting days. Furthermore, not all the 70 registered FBO members may be active in FBO meetings. Nevertheless, we visited the farms we could and recruited 38 out of the 70 registered members. The small sample size limits the generalizability of the study results. Future research can recruit more than one FBO or social group. Again, some of the changes in diet and physical activity patterns, as reported by some participants, could be socially desirable responses, which call for further observational follow-up studies to ascertain the sustained impact of the program. Lastly, we did not assess changes in body weight before and after the intervention. The study duration was short for such an assessment. Future studies with more resources can focus on this assessment.

## Conclusion

The Oklebenor Awareness study implemented a multicomponent community-based intervention to improve knowledge of overweight and obesity. An average of 45 farmers, including men and women, participated in each lesson. The intervention increased participants’ knowledge of overweight and obesity. Secondary outcomes included increased consumption of a four-star diet and physical activity. We recommend integrating culturally sensitive health education approaches with sustained community involvement as an effective strategy for designing and implementing overweight and obesity interventions. We hope these efforts will contribute to a healthier and more informed population, ultimately reducing the burden of overweight and obesity-related health challenges.
